# Do Critical Reviews Affect Box Office Revenues Through Community Engagement and User Reviews?

**DOI:** 10.3389/fpsyg.2022.900360

**Published:** 2022-05-26

**Authors:** Ya-Ling Chiu, Jiangze Du, Yide Sun, Jying-Nan Wang

**Affiliations:** ^1^College of International Business, Zhejiang Yuexiu University, Shaoxing, China; ^2^Shaoxing Key Laboratory for Smart Society Monitoring, Prevention and Control, Shaoxing, China; ^3^School of Finance, Jiangxi University of Finance and Economics, Nanchang, China

**Keywords:** critical reviews, online user reviews, community engagement, box office revenues, electronic word-of-mouth

## Abstract

With advances in technology and the popularity of the Internet, consumers increasingly rely on various sources of electronic word-of-mouth (eWOM), such as online user reviews and critical reviews, in their decision-making processes. Despite general consensus on the importance of eWOM and the ability of critical reviews to influence product sales, very little is known about the mediation between critical reviews and user reviews. Therefore, we used path analysis to examine how critical reviews and user reviews simultaneously affect box office revenues using eWOM data collected from Metacritic.com and IMDb.com, and box office revenue information collected from BoxOfficeMojo.com. The results showed that critical reviews valence not only directly affects box office revenues but also increases active postings in the community and user reviews volume, thus indirectly leading to greater box office revenues. The study provides strategic guidance and practical implications for eWOM communication management.

## Introduction

The popularity of the Internet has clearly affected both the way consumer information is collected and companies’ marketing and communication strategies. Today’s consumers can easily access and use various sources of electronic word-of-mouth (eWOM) information, such as online user reviews (URs) and critical reviews (CRs). In a survey conducted by Podium (2017), an overwhelming 93 percent of consumers agreed that online reviews influence their buying decisions. Accordingly, companies seek to promote their products through online reviews and even obtain information on consumer preferences to develop and improve products. Therefore, online reviews are not only an important reference for consumer decision-making but also an effective marketing communication tool in many industries (Chevalier and Mayzlin, 2006; Liu, 2006; Zhu and Zhang, 2010; Chen et al., 2011; Moe and Trusov, 2011; Zhou and Duan, 2016). The ability to access product evaluation information *via* the Internet reduces consumers’ time and effort to gather information to compare multiple attributes of a product for purchase decisions (Chen and Xie, 2005; Gu et al., 2012). For companies, online product reviews not only reduce marketing communication costs but also accelerate the dissemination of new product information, which in turn affects product sales (Moon et al., 2010; Moe and Trusov, 2011).

Many popular sites, such as Amazon and CNETD, offer both user reviews and critical reviews of listed products. Online user reviews are spontaneously generated by consumers based on their personal experiences, such as movie reviews on IMDb, book reviews on Amazon, restaurant reviews on Yelp, and hotel reviews on TripAdvisor. Online user reviews are considered a valid proxy for WOM and can influence consumer decision-making (Zhu and Zhang, 2010). Many empirical findings support this idea. For example, Godes and Mayzlin (2004) indicated that eWOM volume increases sales in the context of TV shows. Liu (2006) studied movie reviews and found that the information provided by online user reviews has significant explanatory power for box office revenue. Chevalier and Mayzlin’s (2006) study showed that online book ratings affect consumer purchase behavior. In contrast to consumer-generated reviews, critical reviews mainly come from professionals and well-known newspapers, magazines, and media and seek to demonstrate the quality of the product (Chen et al., 2012), such as pre-release movie reviews and professional reviews of selected products on CNETD. Such recommendation information also has a positive impact on the sales of new products. For example, Eliashberg and Shugan (1997) showed that critical reviews are correlated with late and cumulative box office revenue but not with early box office revenue. Thus, they concluded that film critics are predictors rather than influencers. By contrast, Basuroy et al. (2003) showed that professional reviews both influence and predict box office revenue. Similar results were obtained by Boatwright et al. (2007). Regardless of whether they are written by users or critics, online reviews have a positive effect on sales.

Companies are increasingly devoting more resources to eWOM marketing communication because they believe online WOM is far more effective than offline WOM. In addition, many empirical studies have examined the impact of eWOM on sales and generated practical implications (Basuroy et al., 2003; Godes and Mayzlin, 2004; Chevalier and Mayzlin, 2006; Liu, 2006; Duan et al., 2008; Zhu and Zhang, 2010; Zhou and Duan, 2016). However, few studies have discussed whether critical reviews indirectly influence consumer choice through consumer reviewers. We lack an in-depth understanding of the interplay between online critical reviews and user reviews and its influence on consumers’ choices. A previous study by Liu (2006) explored the relationship between the dynamics of online user reviews and movie box office as well as the effect of critical reviews on the volume of user reviews, but this latter effect was not significant. The lack of an effect may be attributable to differences in the sources of WOM, as critical reviews come from physical magazines (i.e., *Variety*), while user reviews come from websites (i.e., *Yahoo! Movie*). As a result, some consumers may not seek out critical reviews. In the context of software downloads, Zhou and Duan (2016) showed that online user reviews mediate the impact of professional reviews on user decisions. This study extends the framework of Zhou and Duan (2016) to investigate the direct impact of critical reviews on the volume of not only user reviews but also postings by community members (i.e., community engagement). In this study, we assume that there is a group of amateur members in the film review community who are passionate about movies and will share their viewing experiences on the platform (not only stat ratings but also text reviews). We expect that the impact of critical reviews on this amateur group of community members will be greater than the impact of critical reviews on the volume of user reviews. In addition, we anticipate that community engagement also indirectly affects box office revenue through the volume of user reviews.

This study collects online review data from IMDb.com, which contains both critical and user reviews, and critical reviews from Metacritic.com, which is a comprehensive source containing a wider range of critical reviews. Data from Metacritic.com has been used in a number of empirical studies (Wiles and Danielova, 2009; Chen et al., 2012; Wang et al., 2015). The purpose of this study is to examine the impact of the mediating role of community engagement in the relationship between critical reviews and user choice. We contribute to the literature in two main ways. First, this study includes two different sources of online reviews (CRs and URs) and examines their direct and indirect effects on consumer choice. Second, an important mediating variable (i.e., community engagement) is proposed in this study. We confirm the mediation model for the effect of CRs and URs on consumer choice, and provide a more complete description of the influence path of eWOM from different sources as well as practical applications. To the best of our knowledge, this work is the first to study the mediating role of community engagement in the relationship between critical reviews and user choices.

## Literature Review and Hypotheses Development

In this study, we draw primarily on two main streams of research in information systems and marketing: (1) the generation of eWOM, and (2) the sales impact of CRs and URs. Based on these related studies, we propose our research model.

### The Generation of Electronic Word-of-Mouth

There are two main theories: psychological motivation theory and review environment theory. Applying these two theories, we propose that CRs can influence the number of URs. Critics and users differ in terms of their motivation and their target audience when writing reviews. Critics write reviews for professional reasons, attempting to avoid emotional and biased statements. Since they write for a wider audience and have considerable experience with quality judgment (Amblee and Bui, 2007). Users, on the other hand, tend to write reviews for personal and emotional reasons (Hennig-Thurau et al., 2004). Another difference is the different timing of posts. CRs usually remain unchanged and are posted earlier than URs, while URs are posted throughout the period of product availability, and such reviews may be influenced by the opinions of other users (Zhou and Duan, 2016). The first theory argues that, in terms of the psychological motivation for users’ sharing behaviors, users are willing to share their experiences and insights in the community is mainly due to self-enhancement (Hennig-Thurau et al., 2004). Self-enhancement refers to the emotional desire to gain attention and improve image among others (Hennig-Thurau et al., 2004). Hennig-Thurau et al. (2004) showed that self-enhancement affects the number of reviews on online opinion platforms. Thus, psychological motivation theory implies that community members are willing to write product reviews. In other words, community members are motivated to post by an intrinsic drive for self-worth reinforcement (Hussain et al., 2018). In the context of movie reviews, online users can consider CRs as an indicator of the attention or popularity of the corresponding movie. In other words, movies that have been reviewed by critics are more visible, compared to those without critical reviews. By posting reviews of movies that have been reviewed by critics through movie sharing platforms, online users may perceive that others are more likely to read their posts, and thus more likely to project themselves as intelligent consumers. On the other hand, online users may anticipate their reviews would be more valuable to other readers in the absence of CRs, and thus be more motivated to write reviews of these movies. Therefore, we expect to see a significant relationship between the CRs and URs. The second theory: review environment theory also supports this relationship between the CRs and URs. Essentially, this theory suggests that environmental factors (i.e., the opinions of others) influence users’ decision to post (Moe and Schweidel, 2012; Goes et al., 2014). The usefulness of information and related interactions are key factors that influence user behavior (Hussain et al., 2020a,b). Similarly, Goes et al. (2014) showed that online user interaction can increase the number of product reviews. Therefore, an increase in the number of consumers participating in the online movie community will increase the number of user reviews (Wu et al., 2018). In addition, when CR is often provided before or at the same time as URs, they help characterize the user’s review environment. Therefore, the evaluation of an individual product by experts can influence the user’s decision whether to review the product or not.

### The Sales Impact of Critical Reviews and User Reviews

**FIGURE 1:**
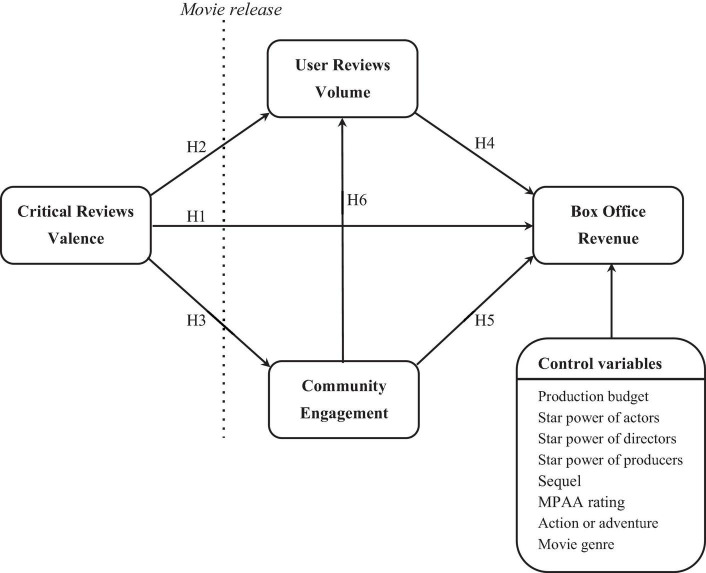
Conceptual framework.

Due to the growing popularity of the Web and social media, CRs and URs now constitute a new element in the marketing communications mix that can have a significant impact on sales (see Table 1 for a summary of representative studies). Two common measures of eWOM have been widely discussed: volume and valence. Volume refers to the number of reviews posted by critics or users. A larger volume of URs can better attract users’ attention to the product/service and accordingly leads to more opportunities to choose it. Previous studies have verified its positive impact on sales (Godes and Mayzlin, 2004; Liu, 2006; Duan et al., 2008; Zhou and Duan, 2016). Valence indicates the favorability of the product, which can be measured by the average ratings of critics or users. Previous studies have examined the impact of URs valence on sales, reporting mixed findings. Some researchers have found a positive impact of URs valence on sales (Chevalier and Mayzlin, 2006; Zhu and Zhang, 2010; Chen et al., 2011). Others have found no significant sales impact of URs valence (Liu, 2006). As another eWOM source, CRs are provided by experts and serve as advertisements (Haan et al., 2005). A relationship between CRs valence and sales has been observed (Eliashberg and Shugan, 1997; Basuroy et al., 2003; Reinstein and Snyder, 2005; Boatwright et al., 2007). Recent studies have begun to investigate the roles of both in the same model (Holbrook and Addis, 2007; Zhou and Duan, 2016). Holbrook and Addis (2007) found that URs valence mediates the effect of CRs valence on popular appeal. Zhou and Duan (2016) showed a similar mediating role of URs, but focused on the URs volume. This study extends work by Zhou and Duan (2016) on the indirect effect of professional reviews on online consumer choices through the volume of user reviews. However, the present study argues that some amateurs are more influential in the film community than others, and when these more influential amateurs recommend or share information about a film in the community, other community members adopt their reviews as a reference for decision-making. Thus, we argue that community engagement is an effective mechanism for influencing consumer choice. We propose that critical reviews influence consumer choice both directly and indirectly through community engagement and online user review volume (as shown in Figure 1). Therefore, this study examines how CRs and URs simultaneously influence consumer choice by focusing on the mediating effects of community engagement.

**TABLE 1:** Literature on the effects of critical reviews (CRs) and user reviews (URs).

Literature	CRs	Effects of CRs	URs	Effects of URs	How CRs and URs Work Jointly	Products
Eliashberg and Shugan, 1997	Variety	CRs valence determines sales.	×	×	×	Movie
Basuroy et al., 2003	Variety	CRs valence affects firm value.	×	×	×	Movie
Reinstein and Snyder, 2005	Siskel and Ebert	CRs valence determines sales.	×	×	×	Movie
Boatwright et al., 2007	Variety	CRs valence determines sales.	×	×	×	Movie
Godes and Mayzlin, 2004	×	×	Usenet	URs volume increases sales.	×	TV show
Chevalier and Mayzlin, 2006	×	×	Amazon, BN	URs valence increases sales.	×	Book
Duan et al., 2008	×	×	Yahoo Movies	URs volume increases sales.	×	Movie
Zhu and Zhang, 2010	×	×	GameSpot	URs volume increases sales. URs valence increases sales.	×	Video game
Chen et al., 2011	×	×	Amazon	URs valence increases sales.	×	Cameras
Moon et al., 2010	Rotten Tomatoes	Ads enhance the effects of CRs valence.	Yahoo Movies	URs valence increases sales. Ads enhance its effectiveness.	×	Movie
Liu, 2006	Variety	CRs valence increases sales.	Yahoo Movies	URs volume increases sales.	×	Movie
Holbrook and Addis, 2007	Rotten Tomatoes	CRs valence affects popular appeal.	IMDb	URs valence affects popular appeal.	URs valence mediates the effect of CRs valence on popular appeal.	Movie
Zhou and Duan, 2016	CNETD	CRs valence affects user choices.	CNETD	URs volume affects user choices.	URs volume mediates the indirect impact of CRs valence on user choices.	Software
This Study	IMDb	CRs valence increases sales.	Metacritic	URs volume increases sales.	URs volume and community engagement mediates the indirect impact of CRs valence on sales.	Movie

### Hypotheses Development

#### Critical Reviews

In recent years, critical reviews have been an important focus of both practical and academic research. Critical reviews provide not only professional reviews to create reputations but also valuable product information for advertising (Haan et al., 2005). Critical reviews often provide a relatively professional critique of the quality of a product and are frequently published before the release of a new product, such as a film. Many studies have shown that critical reviews significantly affect consumer choices and product sales (Eliashberg and Shugan, 1997; Basuroy et al., 2003, 2006; Boatwright et al., 2007). For example, Eliashberg and Shugan (1997) reported that critical reviews are associated with late and cumulative box office revenue but not with early box office revenue. By contrast, Hennig-Thurau et al.’s (2006) study showed that critical reviews have a significant impact on short-term but not long-term box office. In addition, several studies have shown that film critics play dual roles as both influencers and predictors (Basuroy et al., 2003; Boatwright et al., 2007). Reinstein and Snyder (2005) applied a difference-in-difference approach to indicate the impact of movie critics on sales. They found a marginal positive effect of movie critics on demand. Thus, higher critical review valence increases box office revenues (Eliashberg and Shugan, 1997; Basuroy et al., 2003). Therefore, we hypothesize the following:

*H1*: *Critical reviews valence has a positive impact on movie box office.*

Critical reviews have been found to influence the amount of online user-generated review behavior, which is usually measured by the number of online user reviews (Liu, 2006; Duan et al., 2008; Zhou and Duan, 2016). There are three possible reasons for this finding. First, with the development of the Internet and information technology, critical reviews have become widely available in the public domain. The rise of social media has further enhanced the impact of critical reviews on user-generated content (Zhou and Duan, 2016). Second, critical reviews have become a valuable source of information for consumers to evaluate product quality (Chen and Xie, 2005), usually from professional and reputable media or experts in the field (Liu, 2006; Chen and Xie, 2008). Finally, consumers perceive more positive critical reviews as a signal expressing higher popularity and are more willing to share reviews of the corresponding products (Holbrook and Addis, 2007). Past empirical research has found a significant positive correlation between critical reviews and the volume of online user reviews (Holbrook and Addis, 2007; Zhou and Duan, 2016). For example, Holbrook and Addis (2007) reported a positive impact of critical reviews on consumer review volume, and Zhou and Duan (2016) also demonstrated a positive effect of professional reviews on user review volume in the software download context. Hence, we hypothesize the following:

*H2*: *Critical reviews valence has a positive impact on user review volume.*

While Liu (2006) indicated that critical reviews affect consumers’ film expectations and the volume of user reviews, the empirical results did not support this argument. A potential reason is that the critical reviews were obtained from physical magazines (i.e., *Variety*), while the user reviews were obtained from websites (i.e., *Yahoo! Movie*). The use of different data sources may lead to inconsistent results for the impact of critical reviews on the number of user reviews. To overcome this problem, in this study, both critical review and consumer review data were obtained from the website IMDb.com, where consumers can easily access both critical reviews and user reviews of films. In addition, according to review environment theory, consumer comments are influenced by environmental factors, such as the opinions of others (Moe and Schweidel, 2012; Goes et al., 2014). For example, Moe and Schweidel’s (2012) study examined how previously posted product ratings affect an individual’s posting frequency. Their findings show that when the rating environment is positive, consumers are more likely to generate online product reviews after purchase. That is, some amateurs are more willing to engage in the act of posting to the film community if the critical reviews are favorable. Hence, we hypothesize the following:

*H3*: *Critical reviews valence has a positive impact on community engagement.*

#### Online User Reviews

As another eWOM source, online user reviews are product evaluations generated by ordinary users online that mainly provide product quality information based on their purchase experience (Chen and Xie, 2008). There are two types of online user reviews: text product reviews written by customers and numerical star ratings (e.g., between 1 and 5 stars). Both types can be important drivers of product sales and provide consumers with useful product information. In general, text reviews provide richer information than numerical star ratings for consumer decision-making (Salehan and Kim, 2016). In addition, Mudambi and Schuff (2010) suggested that review depth (i.e., the word count of the review) can enhance information diagnosticity and provide increased depth of information to help consumers make decisions with confidence. Chevalier and Mayzlin (2006) pointed out that the length of reviews for a book significantly predicts its sales on Amazon.com. Moreover, the volume of online user reviews (i.e., the total number of user reviews) makes the corresponding product stand out from the crowd and thus gain the attention of consumers (Liu, 2006). Many studies have shown that the number of online user reviews significantly affects consumer choices and product sales (Liu, 2006; Duan et al., 2008; Zhu and Zhang, 2010; Sun, 2012). For example, Liu (2006) showed that online movie review volume has important explanatory power for both total box office and weekly box office revenue. Zhu and Zhang (2010) indicated that online user reviews have a significant positive effect on product sales in the video game industry. Therefore, a large number of user reviews better draws consumers’ attention to the product, increasing the likelihood of choosing that product. Hence, we hypothesize the following:

*H4*: *User reviews volume has a positive impact on movie box office.*

#### Community Engagement

Community engagement is defined as “the consumer’s intrinsic motivations to interact and cooperate with community members” (Algesheimer et al., 2005, p. 21). Consumers share their personal experience, knowledge, and information through the process of actively contributing to the online community, thus highlighting the interactive and bidirectional nature of community participation (Algesheimer et al., 2005). Consequently, many studies suggest that community contribution, such as posting activities, reflects a high level of members’ community participation (Koh et al., 2007) and can be used to measure community engagement (Oestreicher-Singer and Zalmanson, 2013). Community engagement is also related to the desired effect of identification with the brand community on community members. Higher community engagement means higher collaboration and interaction, which in turn will lead to higher positive effects (i.e., community participation or recommendation behavior) (Algesheimer et al., 2005). Recent studies have shown that community engagement is more likely to generate online reviews (Wu et al., 2018), improve online sales (Wu et al., 2015), and enhance customer brand loyalty (Zheng et al., 2015). Hence, we hypothesize the following:

*H5*: *Community engagement has a positive impact on movie box office.*

The opinions shared by consumers in the film community can influence the choices of other community members (Moon et al., 2010). Petty and Cacioppo (1984) argued that the greater WOM, the higher the degree of consumer involvement in products; as a result, consumers are more likely to be influenced by others and shape changes in product attitudes themselves. The more WOM spreads on the Internet, that is, the greater the discussion of the product or service, the more likely WOM is to spread again (Brown and Reingen, 1987; Banerjee, 1992). Goes et al. (2014) showed that users tend to produce more objective reviews in order to gain more trust from their peers. In other words, online reviews have social influence on other consumers (Sridhar and Srinivasan, 2012). When consumers are willing to read and compare user reviews in the movie community, they will obtain more information about the movie. We expect that the depth of the text of reviews will enhance consumers’ ability to detect products, attract more consumer attention, and thereby promote consumers to generate online reviews. As a result, an increase in the number of consumers who engage in the online movie community will increase the number of user reviews (Wu et al., 2018). Therefore, we hypothesize the following:

*H6*: *Community engagement has a positive impact on user review volume.*

## Methodology

### Data Collection Process

To examine whether online user reviews mediate the impact of critical reviews on consumer choice, we collected movie characteristic information, critical reviews, user reviews, and box office using web-scraping techniques. We collected data on the top 100 movie releases by United States box office revenue each year in the approximately 6-year period from January 1, 2010 to December 31, 2015. We obtained movie characteristics such as budget, sequel, genre, MPAA rating, and running time from the Internet Movie Database^1^. We obtained movie box office revenue from Box Office Mojo^2^. After removing 83 movies with incomplete information, we finally included 517 movies that had been successful at the United States box office. Box office revenue per movie ranged from $21.07 million to $933.66 million, with an average of $101.12 million. The data included specific information on movies from six major genre categories: action or adventure (28%), animation (9%), thriller (20%), comedy or drama (46%), sci-fi or fantasy (12%), and romance (15%). Among the included movies, 19% were sequels, and 21% were rated “general” or “parental guidance suggested.” The average production budget was approximately $69.43 million. The maximum production budget was $260 million, and the minimum was $1.5 million; thus, the highest budget was 173 times greater than the lowest. Detailed descriptive statistics for the sample are reported in Table 2.

**TABLE 2:** Descriptive statistics of the attributes of the sample movies within the time period 2010–2015.

Category	Variable	Summary statistics			
Movie characteristics	Sequel	Sequel (98, 19%), Non-sequel (419, 81%)			
	MPAA rating	R (409, 79%), non-R (PG and G) (108, 21%)			
	Six genres	Action or adventure (144, 28%), animation (46, 9%), thriller, horror or crime (101, 20%), comedy or drama (239, 46%), sci-fi or fantasy (61, 12%), romance (75, 15%)			

		**Mean**	**SD*^a^***	**Minimum**	**Maximum**

	*REV* (in millions)	101.12	93.30	21.07 Aloha	936.66 Star Wars: The Force Awakens
	*PB* (in millions)	69.43	58.09	1.50 Insidious	260.00 Tangled
	*SP^A^* (in millions)	149.59	109.45	0.00	609.59 The Butler
	*SP^D^* (in millions)	42.21	79.83	0.00	534.86 Inception
	*SP^P^* (in millions)	128.82	148.20	0.00	978.57 Iron Man 3
Critical reviews	*CRVAL* (range 0–100)	53.72	16.55	11.00 Scary Movie 5	100.00 Boyhood
User reviews	*URVOL*	179071.00	173553.60	6102.00 For Colored Girls	1483904.00 Inception
Community engagement	*POSTVOL*	377.30	390.26	26.00 Diary of a Wimpy Kid: Dog Days	3860.00 Star Wars: The Force Awakens

*^a^an abbreviation of “standard deviation”.*

*REV indicates total box office.*

*PB indicates production budget.*

*SP^A^ indicates star power of actors.*

*SP^D^ indicates star power of directors.*

*SP^P^ indicates star power of producers.*

*MPAA rating indicates whether the movie is rated for age.*

*CRVAL indicates the mean of overall ratings by critical reviews of the movie.*

*URVOL indicates the number of user reviews of the movie.*

*POSTVOL indicates the number of posts about the movie.*

For each movie, critical reviews were collected form Metacritic^3^. Metacritic provides the name of the critic, media outlet, and review score for each movie and each review. We used Metacritic as our primary source of critical review data for the following reasons. First, Metacritic is one of the most comprehensive movie critical review sites, and its coverage and summaries of film reviews have been well-received in the film industry. Second, Metacritic presents a summary of each review in the form of a score on a scale between 0 and 100. These scores provide an independent, professionally assessed index of review valence. Third, movie critical reviews from Metacritic have been increasingly used in recent research (Wiles and Danielova, 2009; Chen et al., 2012; Wang et al., 2015). We also collected film review data from a large number of public online users of IMDb.com. This site was chosen because it is one of the most popular online film review sites, with over 57 million independent visitors per month. More importantly, critical review data from Metacritic can also be found on this site.

### Variable Measurement and Construction

#### Dependent Variable

##### Box Office Revenue

We obtained box office revenue from BoxOfficeMojo.com. Box office revenue (*REV*) was measured as the total box office revenue of a movie in natural logarithm form (Joshi and Hanssens, 2009; Leenders and Eliashberg, 2011). Since the box office revenues were right-skewed, we used a natural log-transformation of the cumulative values, with subsequent skewness and kurtosis values suggesting a normal distribution.

#### Independent Variables

##### Critical Review Valence

The critical review valence (*CRVAL*) was measured by the average score of critics for each movie posted on the Metacritic.com website (Chen et al., 2012).

##### User Review Volume

We obtained user reviews from IMDb.com. User review volume (*URVOL*) was measured by the cumulative number of consumers’ reviews in natural logarithm form (Moe and Trusov, 2011; Sun, 2012).

##### Community Engagement

Community engagement was defined as users’ prosocial contributions to the online movie community. In this study, we measure community engagement (*POSTVOL*) as the number of postings for each movie (Oestreicher-Singer and Zalmanson, 2013; Wu et al., 2018). Higher movie review postings in the community indicated higher customer engagement.

#### Control Variables

We employed some movie characteristics as control variables in this study. First, several studies have shown that the production budget of a movie is significantly related to its box office revenues (Hennig-Thurau et al., 2006; Liu, 2006). Production budget (*PB*) was measured as the production budgets of movies in natural logarithm form (Hennig-Thurau et al., 2006). Second, star power is widely recognized in the film industry (Ravid, 1999). Star power was measured as the sum of the average box office revenue in natural logarithm form for actors (*SP^A^*), directors (*SP^D^*), and producers (*SP^P^*) in the past 3 years, weighted by their ranking among all participants in the same movie production roles. To operationalize star power, we followed Hennig-Thurau et al.’s (2006) suggested method. Taking actors for example, when an actor was listed in the first place on the movie page on Box Office Mojo, the weight given was 1, followed by 0.5, 0.25, 0.125, and so on. Third, sequel movies are generally found to generate higher box office revenues (Basuroy et al., 2006; Moon et al., 2010). *SEQUEL* is a binary dummy taking a value of 1 if the movie was the sequel of a previous movie and 0 otherwise (Hennig-Thurau et al., 2006). Fourth, the MPAA rating is considered an important factor in the industry. Ravid (1999) found MPAA ratings to be significant variables in his research. *MPAA rating* is also a binary dummy variable constructed according to ratings of the appropriateness of movies for different ages as classified by the MPAA: be G (general audiences), PG (parental guidance suggested), PG-13 (parental guidance-13), R (restricted), NC-17 (no one under 17 admitted), and NR (not rated) (Hennig-Thurau et al., 2015). This study categorized G and PG as a group into dummy 1 and PG-13, R, NC-17, and NR as another group into dummy 0. Fifth, genre (*GR*) is a binary dummy constructed according to the classifications used by IMDb, for which the superscripts are *AA*, *AN*, *THR*, *CD*, *SF* and *RO*, representing action, animation, thriller, comedy and drama, sci-fi and fantasy, and romance, respectively (Lee, 2006; Akdeniz and Talay, 2013). The variables, measures, and data sources are reported in Table 3.

**TABLE 3:** Variable definitions, measurements and data sources.

Variables	Definitions	Measurements	Sources	Exemplary studies
*REV*_*i*_	Total box office	The natural logarithm of the total United States box office for movie *i* (USD/in million)	Box Office Mojo*^a^*	Joshi and Hanssens, 2009; Leenders and Eliashberg, 2011
*Critical reviews variable*				
*CRVAL*_*i*_	Critical review valence	Mean of critical reviews published before movie *i* release (range between 0 and 100)	Metacritic*^b^*	Chen et al., 2012; Wang et al., 2015
*User reviews variable*				
*URVOL*_*i*_	User review volume	The natural logarithm of the cumulative number of user reviews for movie *i*	IMDb*^c^*	Moe and Trusov, 2011; Zhou and Duan, 2016
*Community engagement variable*				
*POSTVOL*_*i*_	User posting volume	The natural logarithm of the number of postings to the forum for movie *i*	IMDb*^c^*	Oestreicher-Singer and Zalmanson, 2013; Wu et al., 2018
*Control variables (movie characteristics)*				
*PB*	Production budget	The natural logarithm of the production budget for movie *i* (USD/in million)	Box Office Mojo*^a^*	Hennig-Thurau et al., 2006
*SP^A^*	Star power of actors	The aggregate star power of the actors for the past 3 years. The star power of the actors in a movie is combined with weightings. The weight takes the value 1 if the actor is ranked first, 0.5 if ranked second, 0.25 if ranked third and so forth. The final value is taken in natural logarithm form.	Box Office Mojo*^a^*	Hennig-Thurau et al., 2006
*SP^D^*	Star power of directors	The aggregate star power of the directors for the past 3 years in natural logarithm form.		
				Table 2 continued
*SP^P^*	Star power of producers	The aggregate star power of the producers for the past 3 years. The star power of the producers of a movie is combined with weightings. The weight takes the value 1 if the producer is ranked first, 0.5 if ranked second, 0.25 if ranked third, and so forth. The final value is taken in natural logarithm form.		
*SEQUEL*	Sequel	A dummy variable that takes the value 1 if movie *i* is a sequel and 0 otherwise.	IMDb*^c^*	Hennig-Thurau et al., 2006
*MPAA*	MPAA rating	A dummy variable that takes the value 1 if movie *i* is rated either G (General audiences) or PG (Parental guidance) by the MPAA and 0 otherwise.	IMDb*^c^*	Hennig-Thurau et al., 2015
*GR^AA^*	Action movie	A dummy variable that takes the value 1 if the genre of movie *i* is action or adventure and 0 otherwise.	IMDb*^c^*	Akdeniz and Talay, 2013
*GR^AN^*	Animation movie	A dummy variable that takes the value 1 if the genre of movi*e i* is animation and 0 otherwise.		
*GR^THC^*	Thriller movie	A dummy variable that takes the value 1 if the genre of movie *i* is thriller, horror or crime and 0 otherwise.		
*GR^CD^*	Comedy or drama movie	A dummy variable that takes the value 1 if the genre of movie *i* is comedy or drama and 0 otherwise.		
*GR^SF^*	Sci-fi or fantasy movie	A dummy variable that takes the value 1 if the genre of movie *i* is sci-fi or fantasy and 0 otherwise.		
*GR^RO^*	Romance movie	A dummy variable that takes the value 1 if the genre of movie *i* is romance and 0 otherwise.		

*^a^Box Office Mojo is a website in the United States.*

*It is available at http://www.boxofficemojo.com/.*

*^b^Metacritic denotes the name of a website.*

*It is available at http://www.metacritic.com/.*

*^c^IMDb is an abbreviation of “Internet Movie Database”.*

*The website is available at http://www.imdb.com/.*

## Empirical Analysis

### Correlation Analysis

Table 4 provides a matrix of correlation coefficients for each variable. Box office revenue (*REV*) tended to be positively correlated with critical review valence (*CRVAL*), user review volume (*URVOL*), and community engagement (*POSTVOL*), with correlation coefficients of 0.34, 0.56, and 0.51, respectively. Critical review valence (*CRVAL*) was positively correlated with user review volume (*URVOL*) and community engagement (*POSTVOL*), with correlation coefficients of 0.46 and 0.53, respectively. In addition, a high degree of association was apparent between user review volume (*URVOL*) and community engagement (*POSTVOL*), with a correlation coefficient of 0.86.

**TABLE 4:** Correlation matrix.

Variables	*REV*	*CRVAL*	*POSTVOL*	*URVOL*	*PB*	*SP^A^*	*SP^D^*	*SP^P^*	*SEQUEL*	*MPAA*	*GR^AA^*	*GR^AN^*	*GR^THC^*	*GR^CD^*	*GR^SF^*
*REV*	1.00														
*CRVAL*	0.34***	1.00													
*POSTVOL*	0.51***	0.46***	1.00												
*URVOL*	0.56***	0.53***	0.86***	1.00											
*PB*	0.56***	0.07	0.36***	0.42***	1.00										
*SP^A^*	0.23***	−0.16***	0.01	0.11*	0.44***	1.00									
*SP^D^*	0.21***	−0.09*	0.09*	0.12**	0.23***	0.27***	1.00								
*SP^P^*	0.26***	−0.14**	0.15***	0.15***	0.27***	0.39***	0.27***	1.00							
*SEQUEL*	0.30***	–0.07	0.01	0.05	0.18***	0.07	0.18***	0.10*	1.00						
*MPAA*	0.16***	–0.03	−0.32***	−0.31***	0.17***	0.07	–0.03	–0.05	0.04	1.00					
*GR^AA^*	0.22***	–0.02	0.36***	0.34***	0.40***	0.19***	0.16***	0.16***	0.18***	−0.20***	1.00				
*GR^AN^*	0.27***	0.14**	−0.16***	–0.03	0.24***	0.08	–0.05	–0.05	0.06	0.61***	−0.19***	1.00			
*GR^THC^*	−0.19***	–0.01	0.10*	0.00	−0.29***	−0.16***	–0.07	0.02	–0.03	−0.24***	−0.12**	−0.15***	1.00		
*GR^CD^*	−0.30***	–0.04	−0.30***	−0.21***	−0.33***	–0.08	–0.05	−0.15***	−0.19***	−0.17***	−0.34***	−0.29***	−0.27***	1.00	
*GR^SF^*	0.10*	0.04	0.32***	0.23***	0.24***	0.11*	0.04	0.13**	–0.01	–0.01	0.11*	−0.11**	–0.03	−0.28***	1.00

*REV indicates total box office.*

*CRVAL indicates the mean of overall ratings by critical reviews of the movie.*

*POSTVOL indicates the number of posts about the movie.*

*URVOL indicates the number of user reviews of the movie.*

*PB indicates production budget.*

*SP^A^ indicates star power of actors.*

*SP^D^ indicates star power of directors.*

*SP^P^ indicates star power of producers.*

*SEQUEL indicates whether the movie is a sequel.*

*MPAA denotes whether the movie is rated for age.*

Genre has six categories: action (GR^AA^), animation (GR^AN^), thriller (GR^THC^), comedy and drama (GR^CD^), sci-fi and fantasy (GR^SF^), and romance (base category). ***, **, and * denote significance at the 0.001, 0.01, and 0.05 levels, respectively.

### Path Analysis

**FIGURE 2:**
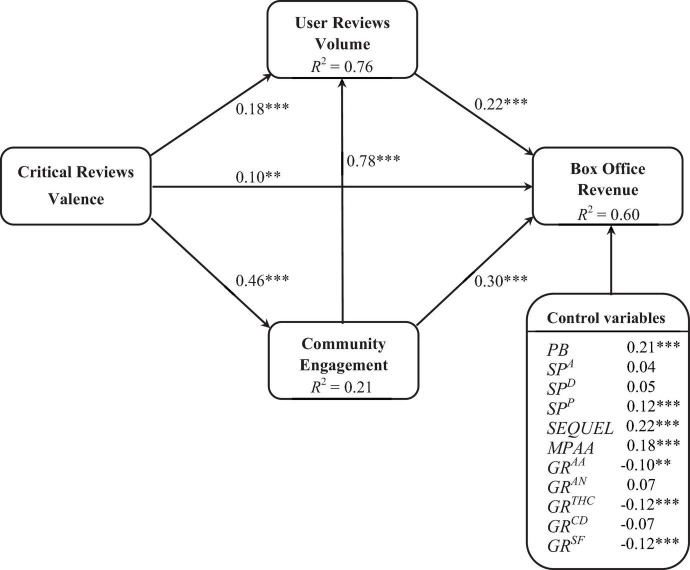
Results of path analysis for the proposed model. ^***^, ^**^ and * denote significance at the 0.001, 0.01 and 0.05 levels, respectively. PB denotes production budget. SP^A^ denotes star power of actors. SP^D^ denotes star power of directors. SP^P^ denotes star power of producers. MPAA rating denotes whether the movie is rated for age. Genre has six categories: action (GR^AA^), animation (GR^AN^), thriller (GR^THC^), comedy and drama (GR^CD^), sci-fi and fantasy (GR^SF^), and romance (base category).

This study investigates a mediation model in which community engagement (*POSTVOL*) and user review volume (*URVOL*) mediate the impact of critical review valence (*CRVAL*) on box office revenue (*REV*). First, as shown in Figure 2, there was a positive effect of critical review valence (*CRVAL*) on box office revenue (*REV*), with a coefficient of 0.10 (*p* < 0.01), supporting H1. Second, critical review valence (*CRVAL*) had positive effects on user review volume (*URVOL*) and community engagement (*POSTVOL*), with coefficients of 0.18 (*p* < 0.001) and 0.46 (*p* < 0.001), respectively, supporting H2 and H3 and indicating a greater impact of critical review valence (*CRVAL*) on community engagement (*POSTVOL*). Third, the direct effect of user review volume (*URVOL*) on box office revenue (*REV*) was significantly positive, with an effect coefficient of 0.22 (*p* < 0.001), supporting H4. Fourth, community engagement (*POSTVOL*) had a positive effect on box office revenue (*REV*), with a coefficient of 0.30 (*p* < 0.001), supporting H5. Fifth, the effect of community engagement (*POSTVOL*) on user review volume (*URVOL*) was significantly positive, with a coefficient of 0.78 (*p* < 0.001), supporting H6. Thus, the more frequent the interaction between community members, the more topics and attention they generate, which in turn creates higher review volume. Finally, at a significance level of 5%, the effect of production budget (*PB)* on box office revenue *(REV*) was significantly positive, echoing findings in the literature (Basuroy et al., 2003; Elberse and Eliashberg, 2003). In addition, critical review valence (*CRVAL*) indirectly affected box office through user review volume (*URVOL*) and community engagement (*POSTVOL*), with an indirect effect coefficient of 0.26^4^.

## Conclusion

This study deepens the understanding of online rating behavior in marketing theory (Chevalier and Mayzlin, 2006; Liu, 2006; Moe and Trusov, 2011; Moe and Schweidel, 2012) by further clarifying how the relationship between CRs and URs affects consumer choice.

### Empirical Findings and Managerial Implications

This study examined whether CRs valence affects product sales through URs processes. The results show that CRs valence may perform better through user review processes such as community engagement and URs volume. These findings further clarify the mechanism by which URs impact the relationship between CRs valence and product sales. This study contributes to the development of theory related to the effect of eWOM on product sales. First, this study shows that CRs valence have a positive effect on sales, consistent with previous findings (Basuroy et al., 2003; Boatwright et al., 2007). We also show that CRs valence has a positive effect on URs volume and community engagement. The present study extends Zhou and Duan (2016) to examine how URs and CRs simultaneously influence online user choices by focusing on the mediation relationship. They pointed out that CRs valence indirectly affect online user choices through URs volume. Consistent with this line of thought, the present study demonstrates that in addition to its direct correlation with URs volume, CRs valence is indirectly related to URs volume *via* community engagement (i.e., postings in the community). The finding is important, as previous research (e.g., Liu, 2006) suggested that CRs valence can affect online users’ expectations of a film and thus influence the URs of the film that are generated. However, this impact may be insignificant if online users do not actually read professional reviews. Conversely, Zhou and Duan (2016) showed that in the software download context, CRs valence has a positive impact on URs volumes. The use of different data sources (i.e., online or offline) may lead to inconsistent results for the impact of CRs valence on URs volumes. To overcome this problem, this study collected both critical and consumer review data from the website IMDb, where consumers can easily obtain both CRs and URs of films. Our study found that CRs valence may also be indirectly related to URs volume through users’ community engagement. Furthermore, we found that the indirect effect of CRs valence on URs volume was greater than its direct effect. This indirect relationship might be due to the fact that users often actively participate in posting in the film community to increase their self-enhancement (Hennig-Thurau et al., 2004). Hennig-Thurau et al. (2004) has shown that self-enhancement has a significant impact on the number of comments on online opinion platforms. Therefore, psychological motivation theory implies that users are willing to write reviews on products.

In addition, we found a marginally significant direct positive relationship between CRs valence and box office revenue when controlling for community engagement and URs volume as intervening variables. The β value of 0.10 indicated partial rather than complete mediation, and the multiplicative indirect effect (0.26) reached significance. Thus, community engagement and URs volume remain important antecedents of box office revenue. The results of previous empirical studies of the effects of CRs valence on box office revenue are inconsistent. Eliashberg and Shugan (1997) showed that professional reviews are related to cumulative box office revenue, whereas Hennig-Thurau et al. (2006) reported that CRs valence have a non-significant impact on long-term box office. The causes of these discrepancies remain to be identified. Our results imply that, due to dilution by mediation *via* community engagement and URs volume, the overall correlation between CRs valence and box office revenue is weak. This finding has implications for the eWOM literature. For example, Holbrook and Addis (2007) concluded that in the case of movie, the relationship between evaluative expert judgment (evaluations of excellence by reviewers) and popular appeal (WOM or market performance) tends to be significant but weak, typically accounting for less than 10% of the variance in popularity or market performance. This study adds to the literature and shows that user posting behavior in the community, in the form of enhanced interaction, is positively associated with box office revenue (β = 0.30, *p* < 0.001; see Figure 2). User posting behavior, together with URs volume and the direct path of CRs valence, accounted for 60% of the variance in box office revenue.

The eWOM plays an important role in the consumer decision making process, and many firms allocate significant marketing budgets to manage their eWOM. Based on our findings, we offer practical implication in social media marketing strategy for movie distributors and studios, as well as provide recommendations for online review platforms. Since we have demonstrated that CRs and URs have different impact effects on sales, those distributors or studios can more strategically prioritize their investments. They may provide incentives for critics or users to focus on different forms of comments and suggestions. In addition, a distributor or studio who hope to increase box office performance could take actions to enhance film community interaction. Potential strategies include enhancing the significance of member participation by ensuring that users understand that their posts could significantly affect the purchase choices of others (Oh et al., 2017). In addition, we strongly urge managers to pay more attention to online communities as an additional monitoring tool for film promotion. By facilitating interactions and communication between potential consumers and existing consumers, user community engagement behavior can increase sales.

Another important practical implication of this study is for the online review platform, our main finding is that in an online world where CRs and URs coexist. CRs can influence URs and further contribute to movie revenues. However, many online review platforms currently rely on user-generated content, and expert reviews are often ignored. In recent years, firms in product categories such as automobiles and electronics have often utilized favorable reviews from professionals and experts on their business information (Eliashberg and Shugan, 1997). In addition, many video game developers use professional reviews to design new products, make sales forecasts, and develop business strategies (Chen et al., 2012). Recent research also claims that CRs remain an influential factor on movie revenues (Basuroy et al., 2020). Therefore, these platforms can design their websites to obtain both types of opinions (i.e., CRs and URs) on the same platform to more effectively influence consumer decisions.

### Limitations and Future Research Directions

This research is subject to limitations that also provide possible directions for future studies. First, this study only considered the volume of online user reviews and did not take into account their valence. The main reason for this choice was that there are no consistent results on the relationship between online user review valence and consumer choice; some studies have shown a positive influence of consumer rating scores on other consumers’ choices (Chevalier and Mayzlin, 2006; Zhu and Zhang, 2010; Chen et al., 2011), whereas others have shown that consumer rating scores do not have a significant effect on consumer choice (Liu, 2006; Baum and Spann, 2014). Some scholars have recently suggested that this discrepancy may be due to the study of the moderation effects of contextual factors (Zhu and Zhang, 2010; Ma et al., 2013). Future discussions and in-depth comparisons could examine whether the impact of consumer ratings varies depending on product type (e.g., books, music) (Zhu et al., 2017) or product popularity (Zhu and Zhang, 2010). Second, we included only eWOM volume and did not analyze review content. It would be worthwhile to explore whether sentiment analysis using a text mining procedure or natural language processing techniques can produce richer consumer information and suggest business strategies (Ludwig et al., 2013; Moon and Song, 2015; Zhu et al., 2017). Textual content can provide additional emotional information about reviews, and different emotional content will have different impact effects (Salehan and Kim, 2016; Ullah et al., 2016), which suggests that integration with textual content can make the eWOM literature more complete and richer. Third, all of the empirical eWOM data were collected from IMDb.com and Metacritic.com. Both websites are focused on the United States, which means that our findings may only reflect the impact of United States movie eWOM on consumer choice. Including data from more websites would allow quantitative measurement and increase the generalizability of the research results.

## Data Availability Statement

The original contributions presented in the study are included in the article/supplementary material, further inquiries can be directed to the corresponding author.

## Author Contributions

All authors listed have made a substantial, direct, and intellectual contribution to the work, and approved it for publication.

## Conflict of Interest

The authors declare that the research was conducted in the absence of any commercial or financial relationships that could be construed as a potential conflict of interest.

## Publisher’s Note

All claims expressed in this article are solely those of the authors and do not necessarily represent those of their affiliated organizations, or those of the publisher, the editors and the reviewers. Any product that may be evaluated in this article, or claim that may be made by its manufacturer, is not guaranteed or endorsed by the publisher.

## Footnotes

1
www.imdb.com

2
www.boxofficemojo.com

3
www.metacritic.com

4 Indirect effect = 0.46 × 0.30 + 0.46 × 0.78 × 0.22 + 0.18 × 0.22 = 0.26.
